# Rheological Behavior and Molecular Interactions in Concentrated Polycarbosilane Solutions in Linear and Cyclic Hydrocarbon Solvents

**DOI:** 10.3390/polym18050550

**Published:** 2026-02-25

**Authors:** Mikhail S. Kuzin, Maria F. Lobanova, Pavel S. Gerasimenko, Maria V. Mironova, Sergey A. Legkov, Ivan Yu. Skvortsov

**Affiliations:** A.V. Topchiev Institute of Petrochemical Synthesis Russian Academy of Sciences, 119991 Moscow, Russia; maria04lobanova@mail.ru (M.F.L.); gerasimenko11507@yandex.ru (P.S.G.); mvmironova@ips.ac.ru (M.V.M.); legkov@ips.ac.ru (S.A.L.); amber5@yandex.ru (I.Y.S.)

**Keywords:** polycarbosilane, decalin, heptadecane, solutions, rheology

## Abstract

Concentrated solutions of polycarbosilane (PCS) are critically important for the development of continuous SiC precursor fibers, where solvent–polymer interactions govern rheology, viscoelastic stability, and spinnability. In this work, PCS solutions in two nonpolar hydrocarbon solvents with different molecular architectures as linear *n*-heptadecane and bicyclic decalin were systematically investigated over a wide concentration range, with emphasis on the semi-dilute entangled and concentrated regimes relevant to solution-based fiber spinning. A combined experimental approach involving steady and oscillatory rheometry and Fourier transform infrared (FTIR) spectroscopy was used to elucidate the influence of solvent structure on solvation, viscoelastic response, microstructural organization, and local intermolecular interactions. Despite similar dilute-solution interaction parameters, the concentrated regimes exhibit pronounced solvent-dependent differences in elasticity and flow behavior. For the first time, linear heptadecane is identified as a viable and technologically promising solvent for PCS, enabling the formation of thermostable homogeneous concentrated solutions with enhanced deformability. This behavior opens a realistic pathway toward a new solution-based fiber-spinning route based on elasticity-controlled processing. The results demonstrate that solvent molecular geometry governs the structure–rheology–processability relationship of concentrated PCS systems rather than solubility parameters alone, providing a new framework for solvent selection in SiC precursor fiber technologies.

## 1. Introduction

Silicon carbide (SiC)-based materials exhibit a unique combination of high strength, hardness, thermal stability, and resistance to aggressive chemical environments, which makes them indispensable for high-temperature and wear-intensive applications [1,2,3,4]. These materials are widely employed in aerospace and rocket engineering, including components of aircraft and propulsion systems, where significant weight reduction and improved performance characteristics can be achieved. The mechanical strength and fracture resistance of SiC-based materials are largely governed by the properties of the reinforcing SiC fibers [5,6,7,8,9].

One approach to producing silicon carbide fibers involves the formation of silicon-containing polymer fibers in films (coats) followed by their subsequent pyrolysis [10,11,12,13,14,15]. In this context, polycarbosilanes (PCS) have emerged as the most important and widely used class of polymeric precursors for SiC fiber production. PCS are typically synthesized via thermal rearrangement and polycondensation of polydimethylsilane or related organosilicon polymers, involving Si–Si bond redistribution reactions that lead to the formation of a Si–C backbone with controlled molecular weight and branching architecture including the synthesis of high-molecular PCS [16]. Recently, approaches using electrochemical synthesis have also been proposed [17]. PCS are valued for their good processability, low oxygen content, relatively small mass loss during pyrolysis, and low free carbon yield, which together enable the formation of high-purity SiC after thermal conversion [18,19,20]. Continuous PCS-derived SiC fibers are therefore widely used as reinforcing elements in ceramic-, metal-, and polymer-matrix composites for high-temperature structural applications, including aerospace components and gas turbine engines [21,22].

The technological foundations of PCS-based fiber production were established by early studies at Tohoku University, which initially focused on direct melt spinning and later extended to drawing fibers from concentrated PCS solutions in benzene. Contemporary processing strategies typically involve melt spinning of PCS followed by oxidative stabilization at 150–200 °C to induce molecular crosslinking, and subsequent pyrolysis up to approximately 1300 °C in an inert atmosphere [23,24,25,26]. As an alternative to oxidative stabilization, electron-beam irradiation with doses on the order of 10–20 MGy has been employed to achieve crosslinking prior to high-temperature conversion [27,28,29].

From a molecular perspective, PCS differ fundamentally from conventional high-molecular-weight linear polymers. They are characterized by relatively low molecular weight, a branched molecular architecture, and a flexible Si–C backbone, which together give rise to complex and non-classical rheological behavior [30]. As a result, both melts and solutions of PCS exhibit atypical viscoelastic responses that are highly sensitive to temperature, concentration, and processing conditions, making rheology a critical factor in fiber-spinning [30].

A major technological challenge in PCS-based fiber production is the high brittleness of precursor fibers prior to pyrolysis, which significantly complicates handling, drawing, and further processing [18]. Despite the central role of the fiber-spinning stage, most existing studies focus primarily on thermal treatment, stabilization, and pyrolysis chemistry, while the hydrodynamics and stability of the polymer jet during melt or solution spinning remain insufficiently explored.

In melt-based processing, fiber thinning and stabilization are governed by a delicate balance between viscous, elastic, and capillary forces. Two limiting failure modes are typically distinguished during jet elongation. Elastic rupture occurs when critical elastic deformation is reached at high extensional rates [31,32,33], whereas capillary breakup is associated with the development of surface instabilities driven by surface tension, viscoelastic stresses, and hydrodynamic pressure gradients [34,35,36,37,38,39,40]. These instabilities directly limit achievable draw ratios and process stability.

An alternative approach to mitigate brittleness and improve processability involves fiber formation from concentrated PCS solutions. Solution-based spinning allows partial decoupling of viscosity and elasticity from temperature, thereby enabling fiber formation at lower effective softening temperatures compared to melt spinning. In this context, the choice of solvent becomes a key parameter, as it determines polymer–solvent affinity, phase behavior, thermal properties of the system, and the balance between viscous and elastic contributions to flow.

The phase state and stability of PCS solutions depend sensitively on solvent structure and compatibility with the polymer chains. Solvent–polymer interactions influence not only solubility and miscibility, but also microstructural organization, the onset of phase separation, and the evolution of viscoelastic properties in concentrated regimes. Recent studies have emphasized that subtle variations in solvent molecular structure may significantly alter intermolecular association and dynamic behavior in organosilicon polymer systems, thereby affecting solution stability and processability [41]. Importantly, existing studies indicate that limited polymer–solvent affinity may, in some cases, be beneficial for fiber spinning, as it promotes intermolecular association and elastic resistance, which can enhance jet stability during elongation.

In the present work, non-polar hydrocarbon solvents were deliberately selected in order to isolate the role of dispersive interactions and steric effects in PCS solutions while avoiding strong specific interactions (e.g., donor–acceptor or hydrogen bonding) characteristic of polar media. The use of polar solvents for PCS is additionally constrained by their potential to introduce oxygen-containing functionalities and promote premature crosslinking or oxidative modifications, which are undesirable for controlled precursor processing. Within the class of non-polar solvents, heptadecane (a linear aliphatic hydrocarbon) and bicyclic decalin were chosen as model systems representing, respectively, flexible linear and conformationally rigid cyclic molecular architectures. This contrast enables a systematic evaluation of how solvent topology affects polymer–solvent affinity, chain packing, and viscoelastic response in concentrated PCS systems. Furthermore, heptadecane possesses a relatively high boiling point, which permits rheological measurements and fiber formation at elevated temperatures without excessive solvent evaporation, thereby significantly expanding the accessible processing window and enhancing the flexibility of technological parameter optimization.

Rheology therefore represents a central tool for understanding and controlling the stability of PCS fiber spinning. Both shear and extensional rheological properties govern jet thinning, resistance to breakup, and the transition from liquid-like to solid-like behavior during spinning. Extensional rheology of polymer jets, combining optical observations, mechanical stress measurements, and theoretical modeling, has been increasingly applied to analyze the stability of viscoelastic jets and the conditions leading to elastic or capillary failure [35,38,42,43,44,45,46]. Although these approaches are still under development, they provide critical insight into the mechanisms governing stable fiber formation and scalability.

Despite the recognized importance of solvent effects, a systematic comparative analysis of linear and cyclic hydrocarbon solvents in relation to solvent–polymer affinity, intermolecular association in concentrated PCS systems, and the resulting shear viscoelastic response remains lacking. The scientific novelty of the present work lies in establishing a structure–property relationship between solvent molecular architecture and the rheological behavior of concentrated PCS solutions, integrating FTIR-based assessment of specific intermolecular interactions with quantitative rotational rheometry. This approach enables identification of rheological signatures associated with enhanced elastic resistance and improved jet stability, thereby providing a framework for solvent selection in solution-based spinning of PCS precursor fibers.

In this context, the present study is aimed at a systematic investigation of the solvent–polymer interactions and rheological properties of concentrated PCS in linear and cyclic hydrocarbon solvents. By combining rotational rheometry, and FTIR, this work seeks to elucidate how solvent molecular structure influences solubility, viscoelastic response, and flow stability in concentrated PCS systems. The ultimate goal is to identify solvent systems and rheological regimes that are most favorable for stable solution-based spinning of PCS precursor fibers, thereby providing a rational basis for reducing precursor brittleness during spinning and improving the scalability of SiC fiber production.

## 2. Materials and Methods

### 2.1. Materials

The polycarbosilane used in this study was supplied by JSC “GNIHTEOS” (Moscow, Russia). The main physicochemical characteristics of the polymer are summarized in Table 1. The material is characterized by a relatively low molecular weight, moderate polydispersity, and a high hydrogen content, which are typical for PCS grades used as SiC precursors.

Decalin and heptadecane were used as solvents. Both solvents were purchased from ECOS-1 (Moscow, Russia). Their key physicochemical and thermal properties are summarized in Table 2.

### 2.2. Methods

#### 2.2.1. Solution Preparation

**Figure 1:**
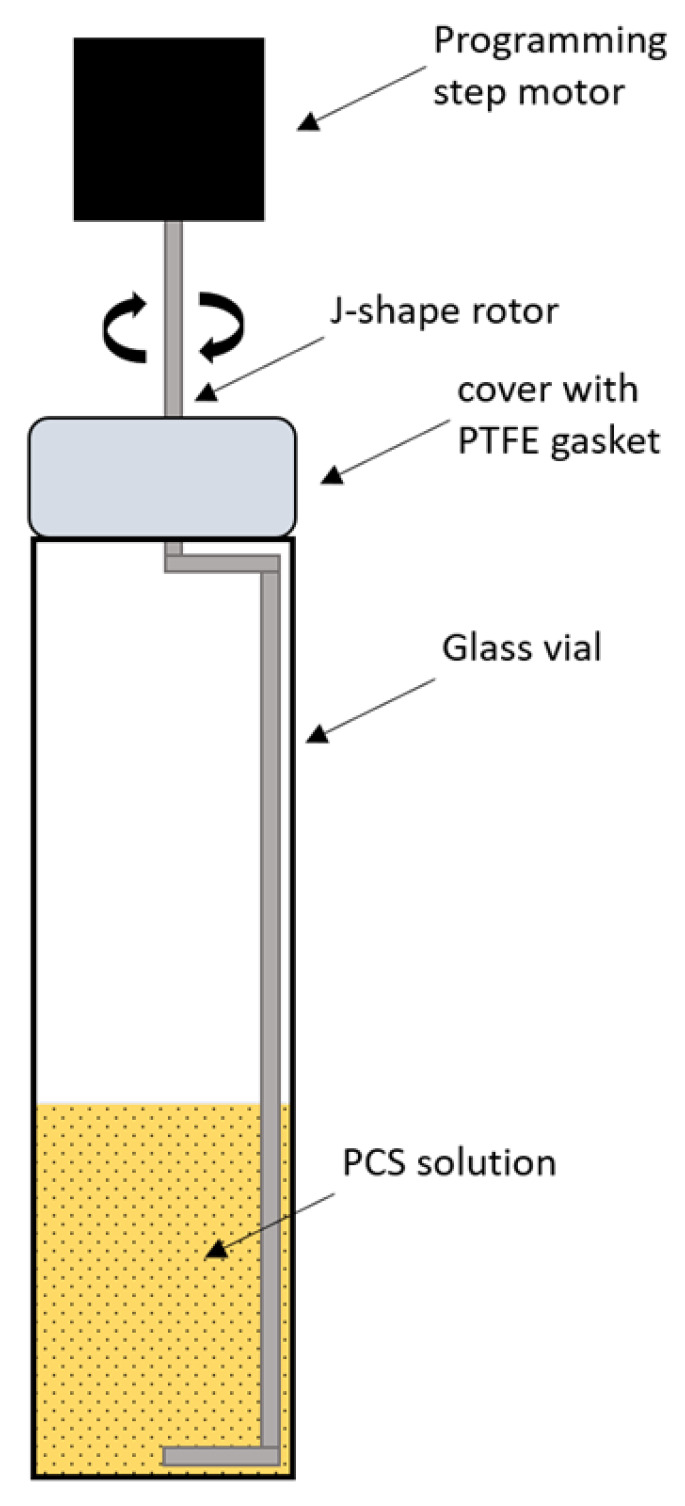
Scheme of J-shaped rotor mixing.

Polycarbosilane powder and solvent were mixed in glass vials with hermetically sealed lids. Different dissolution methods were employed based on the polymer concentration:

High-viscosity solutions with a polymer content greater than 5 wt% were prepared using a paddle mixer with a J-shaped rotor (Figure 1). Mixing was conducted at 60 rpm for 24 h at 70 °C.

#### 2.2.2. Intrinsic Viscosity

Intrinsic viscosity [*η*] of all solutions under study was obtained as per ASTM D2857 with an Ubbelohde capillary viscometer at 25 °C [47]. The experimental data were processed following the standard extrapolation procedure based on measurements at several concentrations.

Sequential dilution was carried out directly in the Ubbelohde viscometer by adding a precisely measured volume of solvent to the initial solution. After each addition, the mixture was homogenized by gentle rotation of the viscometer, followed by five preliminary flow cycles through the measuring capillary to ensure complete mixing and removal of flow history effects. The system was then thermostated for 10 min at 25 °C prior to each viscosity measurement to ensure thermal equilibrium.

#### 2.2.3. Rheology

Systematic rheological estimation of all solutions under study was performed using a HAAKE MARS 60 rotational rheometer (Thermo Fisher Scientific, Germany, Karlsruhe). Experimental data were obtained using the “cone-plate” geometry of the measuring unit with diameters of 20 and 60 mm and the angle between conical and plate surfaces equal to 1 deg. For solvents and extremely low-viscosity solutions, the biconic measuring unit was used at 60 mm, with the angle between conical and plate surfaces equal to 1 deg.

A protective cup was installed during the experiment to prevent evaporation. Moreover, the sides of the measuring unit were covered using a polydimethylsiloxane liquid.

Flow curves for all solutions under study were measured at a steady regime of shearing in the range from 0.01 to 1000 s^−1^.

The viscoelastic properties of solutions were initially measured for identifying the limit of linearity in the range of amplitudes from 10^−3^ to 10 rel. units (0.1–1000%) at two constant angular frequencies of 1 (6.3 rad/s) and 80 Hz (503 rad/s). Then, the frequency dependencies of the components of the complex modulus–storage modulus $G^{\prime }(\omega )$ and loss modulus $G^{\prime \prime }(\omega )$ were measured in the frequency range from 0.628 to 628 rad/s in the linear domain of the viscoelastic behavior.

#### 2.2.4. FTIR Spectroscopy

The FTIR spectra of all samples were obtained in reflection mode from the surface using an IFS-66v Bruker Fourier IR spectrometer (Bruker, Billerica, MA, USA) with Attenuated Total Reflectance (ZnSe crystal, 50 scans, resolution 2 cm^−1^, range 4000–600 cm^−1^).

## 3. Results

### 3.1. Interaction of Polymer and Solvent

**Figure 2:**
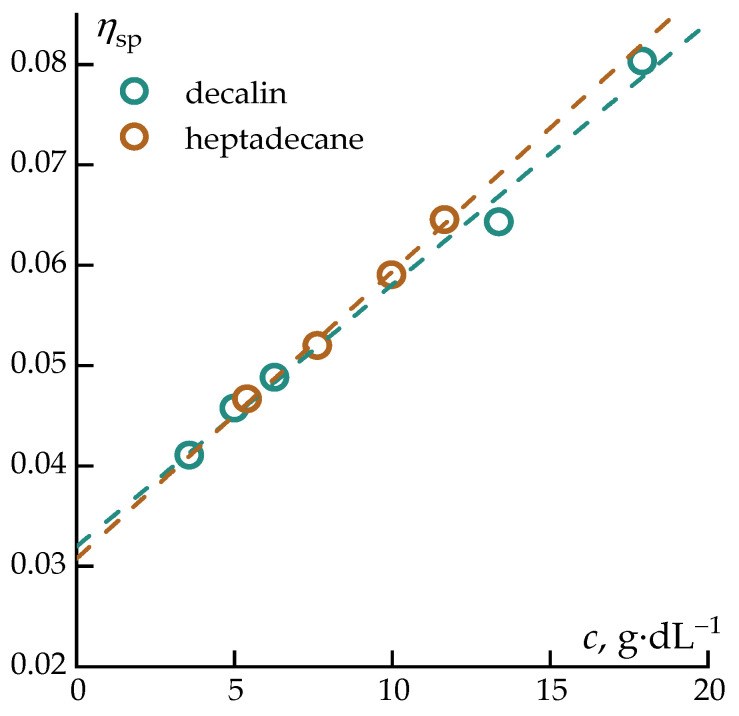
Dependence of the reduced viscosity on the concentration of the PCS solution in various solvents in the coordinates of the Huggins equation.

The interaction between polycarbosilane and the selected solvents was first analyzed in the dilute solution regime using capillary viscometry. Figure 2 presents the concentration dependences of the reduced viscosity of PCS solutions in decalin and heptadecane, plotted in the coordinates of the Huggins equation [48] (Figure 2). This representation enables a quantitative assessment of hydrodynamic behavior and polymer–solvent interactions within the limits of isolated macromolecular coils.

The experimental data were analyzed using the classical relation:

$$\frac{\eta_{sp}}{c}=\left[\eta \right]+k_{H}c{\left[\eta \right]}^{2}$$

where $\eta_{sp}$ is the specific viscosity $\eta_{sp}=\frac{\eta -\eta_{s}}{\eta_{s}}$, η is the viscosity of the polymer solution, $\eta_{s}$ is the solvent viscosity, c is the polymer concentration in g/dL, $k_{H}$ is the Huggins constant, and $\left[\eta \right]$ is the intrinsic viscosity.

The intrinsic viscosities of PCS in both solvents were identical within experimental error [*η*] = 0.03 ± 0.01 dL·g^−1^, indicating that the hydrodynamic volume of PCS macromolecules and the effective coil dimensions are practically independent of the nature of the solvent in the studied systems. This result suggests a very similar solvent quality for both decalin and heptadecane with respect to PCS, despite their different molecular structures (bicyclic vs. linear n-alkane).

It should be noted that the intrinsic viscosity of PCS with the indicated molecular weight is somewhat lower than that of linear PEG of the same molecular weight ([η] = 0.048 dL·g^−1^ for PEG-1000) [49], which may indicate a probable branched architecture of PCS, similar to that observed for branched PAN in solution [50].

However, viscometric analysis provides only indirect information on the nature of intermolecular interactions. In order to identify possible specific chemical interactions (e.g., weak donor–acceptor interactions, Si–H···H–C contacts, or solvation effects involving Si–C and Si–H groups) and to clarify the molecular origin of the observed non-typical viscosity constants, further analysis based on FTIR spectroscopy of PCS solutions was performed.

### 3.2. FTIR Spectroscopy

**Figure 3:**
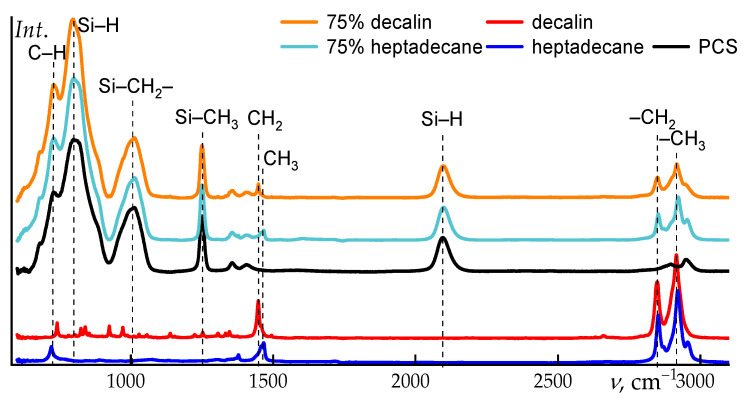
IR spectra of decalin, heptadecane, and PCS.

Figure 3 shows the spectra of the initial PCS and its solvents, decalin and heptadecane.

**Figure 4:**
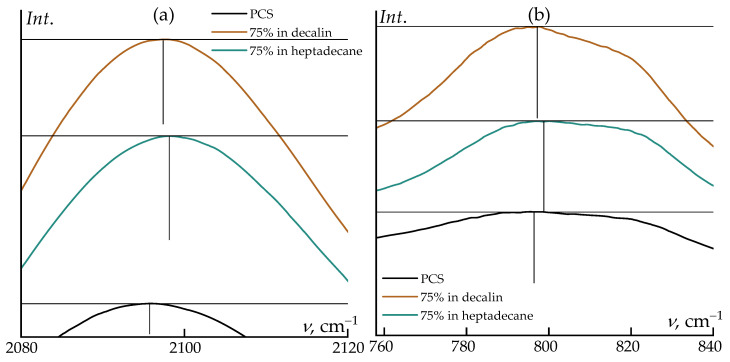
FTIR spectra of PCS and its solutions in decalin and heptadecane: (**a**) region of ~2100 cm^−1^ corresponding to the stretching vibrations ν(Si–H); (**b**) region of ~800 cm^−1^ assigned to the bending (deformation) vibrations δ(Si–H).

The absorption bands of PCS and the employed solvents are well separated, as evidenced in Figure 4. This spectral separation enables a detailed analysis of structural and conformational changes in the polymer upon dissolution. In the PCS spectrum, the most pronounced features include the deformation vibrations in the region around 800 cm^−1^, assigned to Si–H_2_ modes, as well as the asymmetric stretching vibrations of the Si–CH_2_–Si moieties near 1010 cm^−1^, the 1250 cm^−1^ band for Si–CH_3_ stretching, and the Si–H stretching vibration at 2100 cm^−1^. In addition, the valence vibrations of terminal –CH_3_ groups are clearly resolved.

Notably, changes in the relative intensities of the bands at ~795 cm^−1^ and the shoulder near ~820 cm^−1^ are observed: in decalin, the 795 cm^−1^ band becomes dominant, whereas in neat PCS both features exhibit comparable intensities. A slight frequency shift of the ~1013 cm^−1^ band (~1 cm^−1^) is also detected in decalin.

Upon transition from neat polycarbosilane to its concentrated (75 wt%) solutions in decalin and heptadecane, systematic shifts of several characteristic bands are observed in the IR spectra. The most pronounced changes are associated with the Si–H stretching vibration (~2096 cm^−1^) and the coupled deformation–stretching mode of Si–C/Si–CH_3_ (~796 cm^−1^). Dissolution leads to a shift of the Si–H band towards higher wavenumbers, reaching 2097.4 cm^−1^ in decalin and 2097.8 cm^−1^ in heptadecane. Similarly, a moderate upshift is observed for the ~796 cm^−1^ band, which moves to 797.2 and 798.7 cm^−1^, respectively.

These spectral changes are not associated with any chemical transformations and exclusively reflect the influence of the local microenvironment. In the condensed PCS phase, interchain dispersive interactions slightly “soften” the Si–H and Si–C vibrational modes. Upon dissolution, the packing density decreases, the local polarizability of the medium is reduced, and specific interactions are essentially absent. As a result, the corresponding vibrational modes become effectively stiffer, giving rise to a moderate high-frequency shift on the order of 1–3 cm^−1^.

The differences between decalin and heptadecane are also consistent with their physicochemical properties. Linear heptadecane provides a slightly less polarizable microenvironment compared to cyclic decalin, which leads to a somewhat larger frequency increase for both the Si–H stretching band and the mode near 796 cm^−1^. At the same time, the positions of the remaining bands (CH_2_/CH_3_ vibrations and low-frequency skeletal modes) remain essentially unchanged, confirming the absence of any structural rearrangements of the polymer backbone upon dissolution.

### 3.3. Rheology of Concentrated Systems

**Figure 5:**
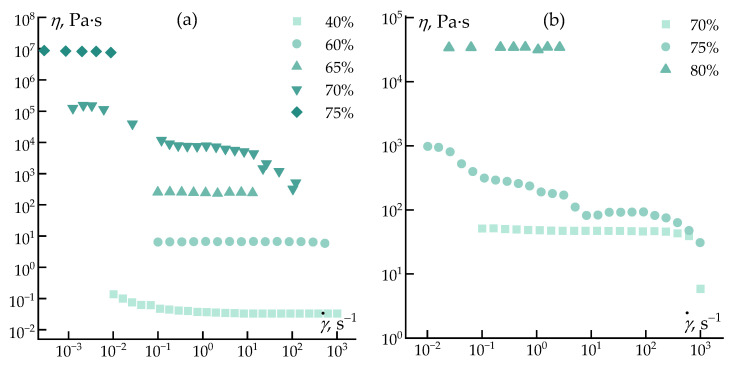

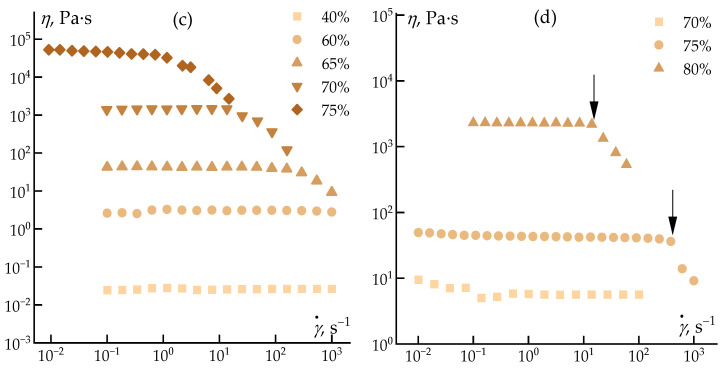
Dependence of the viscosity of the PCS solution in decalin at 25 °C (**a**) and at 70 °C (**b**) and in heptadecane at 25 °C (**c**) and at 70 °C (**d**). The arrows show the beginning of the spurt.

To elucidate the influence of solvent nature on the rheological behavior of polycarbosilane solutions, rheological measurements were performed over a wide concentration range. The steady shear flow curves of PCS solutions in decalin and heptadecane at 25 and 70 °C are presented in Figure 5.

As shown in Figure 5, at low polymer concentrations the PCS solutions in both solvents exhibit nearly Newtonian behavior, with viscosity being weakly dependent on shear rate. In this concentration regime, the viscosity of the solution is primarily governed by the viscosity of the solvent itself, while polymer–polymer interactions remain negligible. Consequently, the absolute viscosity values in this region reflect differences in the intrinsic viscosities of decalin and heptadecane rather than the contribution of the polymer.

With increasing PCS concentration, a gradual transition to non-Newtonian, shear-thinning behavior is observed, which is characteristic of viscoelastic polymer solutions. In the semi-dilute and concentrated regimes, polymer–polymer interactions become dominant, leading to the formation of a transient network of entanglements that governs the flow response. For PCS solutions in heptadecane, the onset of shear thinning occurs at lower concentrations and at lower shear rates compared to decalin-based systems. This behavior indicates an earlier development of intermolecular associations and a less efficient solvation of PCS chains in the linear hydrocarbon solvent.

In contrast, PCS solutions in decalin exhibit more extended Newtonian plateaus and a smoother transition to shear-thinning flow, particularly at elevated temperature (70 °C). This suggests that the cyclic structure of decalin provides more effective screening of polymer–polymer interactions, resulting in a more homogeneous stress distribution and delayed formation of a strongly interconnected polymer network. Increasing temperature leads to a systematic reduction in viscosity for both solvent systems; however, the qualitative differences between decalin and heptadecane persist, indicating that solvent molecular structure plays a decisive role beyond purely thermal effects.

**Figure 6:**
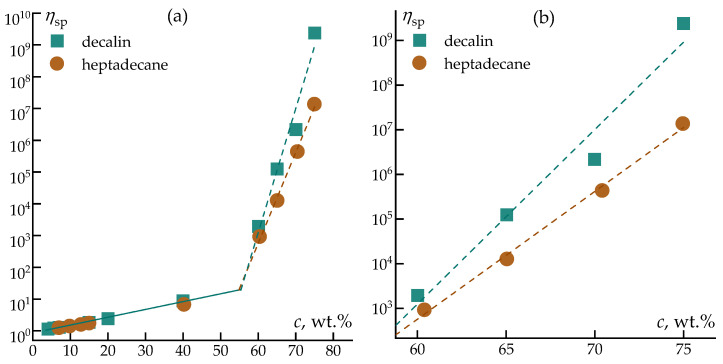
Dependence of specific viscosity on the volume occupied by a polymer coil in a wide range (**a**) and in concentraded range (**b**) of a PCS solution in various solvents at 25 °C.

To obtain a generalized view of concentration effects, the values of zero-shear (Newtonian plateau) viscosity were used to construct the dependence of specific viscosity on the parameter characterizing the volume occupied by the polymer coil (Figure 6).

As shown in Figure 6, the concentration dependence of specific viscosity for PCS solutions in both solvents exhibits the characteristic behavior of polymer solutions across diluted, semi-dilute, and concentrated regimes. At low values of the coil volume parameter, corresponding to dilute solutions, the increase in specific viscosity is modest and primarily reflects hydrodynamic contributions of isolated macromolecules. Upon further increase in concentration, a pronounced growth in specific viscosity is observed, indicating the onset of chain overlap and the formation of an entangled polymer network.

At comparable values of the polymer coil volume parameter, PCS solutions in heptadecane display higher specific viscosities than those in decalin. This observation confirms that polymer–polymer interactions emerge at lower effective concentrations in heptadecane, consistent with reduced solvent affinity. In contrast, the shift of the rapid viscosity increase toward higher concentrations for decalin-based solutions indicates more efficient solvation of PCS chains and delayed network formation.

Overall, despite the chemical similarity of decalin and heptadecane, differences in their molecular architecture led to pronounced changes in the rheological response and concentration regimes of PCS solutions. These differences directly affect the formation of viscoelastic networks, flow stability, and resistance to deformation, which are critical factors for stable jet thinning and fiber formation. The presented results highlight rheology as a key parameter for evaluating solvent suitability in solution-based processing of polycarbosilane precursor fibers.

**Figure 7:**
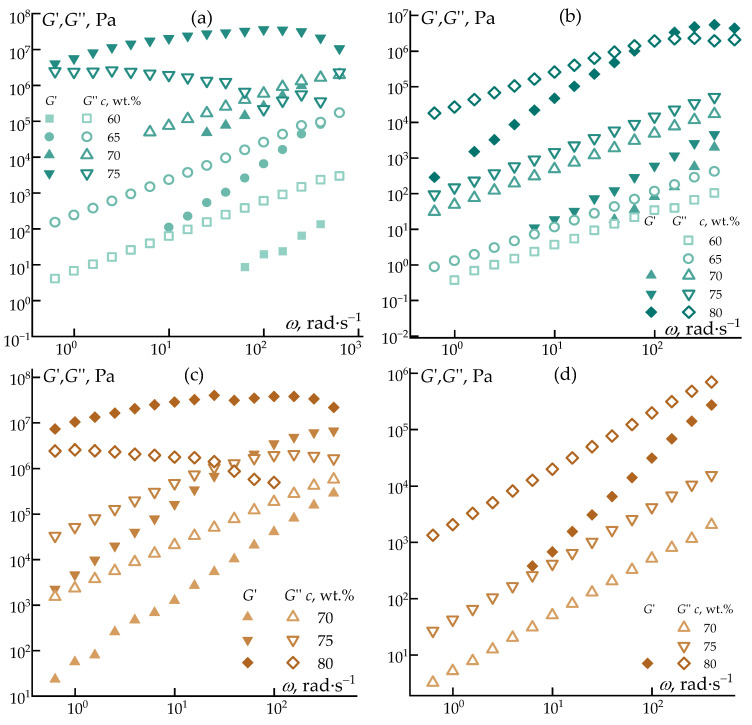
Frequency dependences of elastic moduli and loss moduli on the concentration of the polymer solution in decalin at 25 °C (**a**) and 70 °C (**b**) and in heptadecane at 25 °C (**c**) and 70 °C (**d**).

Figure 7 shows the frequency dependences of the loss and accumulation moduli.

Regardless of the solvent nature, all solutions exhibit behavior characteristic of polymer systems, namely a systematic increase in viscoelastic moduli with increasing polymer concentration. In all cases, the growth of the elastic response is more pronounced than that of the viscous contribution and is accompanied by a distinct change in the slope of the modulus–frequency curves, indicating a deviation from the ideal Maxwell model. This behavior reflects the increasing contribution of intermolecular interactions and the progressive formation of transient elastic networks within the solution.

It should be noted that for several intermediate-temperature systems (e.g., 60 and 65 °C in Figure 7b, as well as 70 and 75 °C in Figure 7d), reliable G’ values could not be obtained. At these temperatures, the corresponding solutions exhibited extremely weak elastic response, with G′ approaching the lower sensitivity limit of the rheometer. Under such conditions, the measured storage modulus becomes strongly affected by instrumental noise and torque resolution limits, leading to non-reproducible and physically inconsistent values. These data were therefore excluded from the figures to avoid misinterpretation, as they do not represent reliable viscoelastic characteristics of the systems.

For some systems, particularly for highly concentrated solutions (75 and 80 wt.%) at 25 °C, an inflection point was observed on the elastic modulus curves, suggesting a qualitative change in the dominant relaxation mechanisms. This feature indicates a transition toward a more structured viscoelastic state, associated with restricted chain mobility and the emergence of long-lived relaxation modes.

The temperature dependence of relaxation processes is well described by the Williams–Landel–Ferry (WLF) equation (Equation (1)) [51]:

(1) $$\log\alpha_{T}=-\frac{C_{1}\left(T-T_{0}\right)}{C_{2}+\left(T-T_{0}\right)}$$

where α_T_ is the temperature shift factor, T is the current temperature, T_0_ is a reference temperature, and *C*_1_ and *C*_2_ are constants related to the free volume and the rate of its variation with temperature.

The shift factor α_T_ can be determined either from the ratio of relaxation times τ or from the ratio of viscosities measured at two temperatures, according to Equation (2):

(2) $$\alpha_{T}=\frac{\tau \left(T\right)}{\tau \left(T_{O}\right)}=\frac{\eta \left(T\right)}{\eta \left(T_{O}\right)}$$

**Figure 8:**
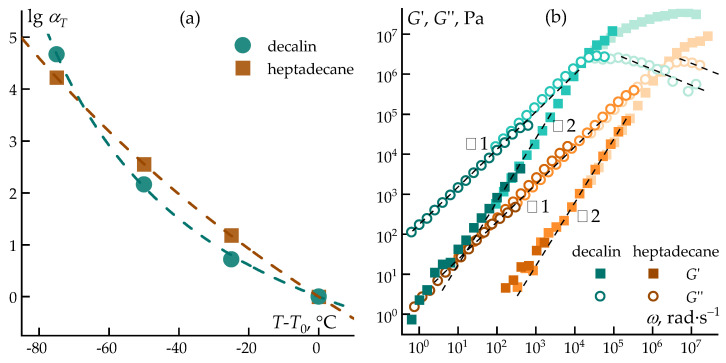
Dependence of the shift coefficient on temperature: the dots represent the experimental data, and the dashed curve represents the theoretical model (**a**) and the general TTS curve (**b**) of the storage moduli G’ (open symbols) and loss moduli G” (filled symbols) as functions of the angular frequency ω. The dashed curves indicate compliance with the Maxwell model.

Using the results of rheological measurements, the temperature shift factors (*α*_*T*_) were calculated, and a comparison between experimental values and those predicted by the WLF equation is shown in Figure 8a. To extend the analysis of viscoelastic properties over a broader frequency range, the principle of time–temperature superposition was applied, and the corresponding master curves are presented in Figure 8b. The viscoelastic behavior of PCS solutions in both solvents shows good agreement with the Maxwell model, as evidenced by the slopes of the modulus–frequency dependencies, with characteristic tangent values of 1 and 2 corresponding to the terminal and elastic regimes [52], respectively.

## 4. Conclusions

By combining rheological characterization and infrared spectroscopy, it is shown that even in chemically similar nonpolar hydrocarbon media, the distinction between linear and cyclic solvent structures leads to fundamentally different solution microstructures and flow regimes in the concentrated state.

Although dilute solution parameters (intrinsic viscosity) indicate equal atypical polymer–solvent interactions for both systems, the concentrated regimes reveal clear qualitative differences. Cyclic decalin favors more compact solvation and hydrodynamic stabilization of PCS chains, resulting in structurally stable but mechanically less deformable systems. In contrast, linear heptadecane forms concentrated PCS solutions with enhanced elasticity, higher compliance, and increased deformational capacity, despite less efficient molecular solvation.

Most importantly, this work demonstrates for the first time that heptadecane is not only an applicable solvent for polycarbosilane but also a technologically promising medium for solution processing. The formation of highly elastic, concentrated PCS solutions in heptadecane opens a promising new direction for the development of solution-based spinning routes, where elasticity-dominated rheology can be exploited to improve drawability, reduce fiber brittleness, and expand the processing window for continuous fiber spinning.

These findings provide a foundation for further studies aimed at correlating solvent geometry with spinnability, fiber morphology, and microstructure evolution during thermal conversion to SiC. Moreover, the demonstrated suitability of heptadecane and decalin opens up the possibility of designing joint PCS–Polyolefin for the fabrication of composite materials and their subsequent application in additive manufacturing technologies, where controlled viscoelastic behavior is critical for shape retention and structural integrity during processing.

At a fundamental level, the results establish that solvent geometry (linear vs. cyclic), rather than classical solubility parameters alone, governs the balance between solvation, chain packing, elastic network formation, and flow stability in concentrated PCS systems. This introduces a new design principle for solvent selection in PCS spinning technologies, shifting the focus from purely thermodynamic compatibility toward structure-controlled rheological engineering.

## Figures and Tables

**Table 1:** Physicochemical properties of polycarbosilane used in this work.

Property	PCS
Carbon content (C), wt.%	40.39
Silicon content (Si), wt.%	8.60
Hydrogen content (H), wt.%	50.76
Hydrogen bound to silicon (HSi), wt.%	0.68
Softening temperature (ring-and-ball method), °C	238
Number-average molecular weight (*M*_n_), g·mol^−1^	1250
Weight-average molecular weight (*M*_w_), g·mol^−1^	3200
Polydispersity index (*PDI* = *M*_w_/*M*_n_)	2.5

**Table 2:** Physicochemical and thermal properties of the solvents used in this study.

Property	Decalin (C_10_H_18_)	Heptadecane (C_17_H_36_)
Molecular structure	Bicyclic saturated hydrocarbon (cis-/trans-mixture)	Linear saturated n-alkane
Molecular weight, g·mol^−1^	138.25	240.47
Density at 25 °C, g·cm^−3^	~0.89	~0.78
Boiling point, °C	~187–195	~302
Melting point, °C	−43 to −30	~22
Dynamic viscosity at 25 °C, mPa·s	~2.5–3.0	~3.5–4.0
Vapor pressure at 25 °C	Moderate	Very low

## Data Availability

The raw data supporting the conclusions of this article will be made available by the authors on request.
